# The Influence of Copper Substrates on Irradiation Effects of Graphene: A Molecular Dynamics Study

**DOI:** 10.3390/ma12020319

**Published:** 2019-01-21

**Authors:** Shulong Wang, Qian Zhang, Kai Yin, Bo Gao, Siyu Zhang, Guoping Wang, Hongxia Liu

**Affiliations:** 1 Key Laboratory for Wide Band Gap Semiconductor Materials and Devices of Education, School of Microelectronics, Xidian University, Xi’an 710071, China; qian_one@126.com (Q.Z.); malong_gw@163.com (K.Y.); 13201517636@163.com (S.Z.); crystal-one@outlook.com (G.W.); 2 Xi’an Institute of Microelectronics Technology, Xi’an 710000, China; 15029075229@163.com

**Keywords:** graphene, copper substrate, MD simulation, defects

## Abstract

In this paper, classical molecular dynamics simulations are conducted to study the graphene grown on copper substrates under ion beam irradiation, in which the emphasis is put on the influence copper substrate on a single graphene layer. It can be inferred that the actual transmission and distribution of kinetic energy from incident ion play important roles in irradiation-defects forming process together. The minimum value needed to generate defects in supported graphene is higher than 2.67 keV, which is almost twice the damage threshold as the suspended graphene sheet. This work indicates the presence of copper substrate increases the damage threshold of graphene. Additionally, our results provide an atomistic explanation for the graphene with copper substrate under ion irradiation, which is very important for engineering graphene.

## 1. Introduction

Graphene is a one-atom-thick planar sheet consisted of sp2-bonded carbon atoms arranged in a honeycomb crystal lattice [1]. As graphene has novel electronic properties (the electron mobility is 2 × 10^5^ cm^2^·v^−1^·s^−1^), optical properties (the transparency is 97.9%), thermal Properties (the thermal conductivity is 5000 W·m^−1^·K^−1^), mechanical properties (the Young’s modulus is 1.1 TPa), and the 2630 m^2^/g specific surface area, it is considered to be a promising candidate for application of various fields, such as electronic components, photons sensors, thermal materials, and gene sequencing [2,3,4].

In order to realize the applications of graphene, a significant amount of fundamental research is dedicated towards controllable modification of the properties of graphene, especially the method for accurately changing the atomic structure and thereby affecting the electronic properties of the material, which is usually obtained by introducing doping into the material. For graphene, the defects introduced in the crystal lattice can transform the semi-metal properties without forbidden band into a band-tunable semiconductor [5,6,7]. Current doping methods often require gases and precursors, and require relatively high temperatures to undergo chemical reactions [8,9]. These methods have a common shortcoming, which is that they mostly require high temperatures, precursors, and multiple gases, which can cause uneven doping and can even cause contamination of the sample. The irradiation method can precisely control the doping and defect generation, and at the same time avoid these problems. Therefore, the method of modifying graphene based on different irradiation sources has been widely studied [10,11,12,13,14,15]. The main sources of radiation are ionizing ions, electron beams, lasers, X-rays, gamma rays, microwaves, etc. These radiation sources can achieve precise modification of graphene under certain conditions, and have thus been extensively studied. 

In this regard, irradiation is a promising tool in doping graphene with foreign atoms that has been used to introduce various defects in graphene in order to engineer its properties [16,17]. Until now, most of the researches are focused on the irradiation effect on suspended graphene layer [18,19,20]. However, the effect from the substrate underneath the graphene is also found playing an important role in the defect formation process. Hence, in this work, the molecular dynamics (MD) simulations are performed to research the influence of the copper substrate on the irradiation effect of single layer graphene (SLG), which are well known to be the best method for studying the irradiation processes on an atomistic level [16,17,21]. In order to consider the bonding interaction effects between irradiated ions and atoms in graphene, carbon ions are chosen as irradiated ions, which have been demonstrated to be experimentally feasible.

## 2. Materials and Methods 

For the simulation of the ion-solid interaction process, this work used classical MD simulation in order to carefully investigate the mechanism of the defect formation in graphene by using the software Large-scale Atomic/Molecular Massively-Parallel Simulator (LAMMPS, Sandia National Laboratories, Livermore, CA, USA). Additionally, periodic boundary conditions are imposed in both the x- and y-directions. The simulation model of graphene consists of 963 carbon atoms with the dimensions around 5 nm × 5 nm. The thickness of copper substrate is 10 nm. The incident area around the center of graphene within 2.5 nm radius. The adaptive intermolecular reactive bond-order (AIREBO) potential function is used to model the interactions between carbon atoms (C–C) in graphene. In addition, this potential has also been successfully used to simulation the irradiation effects of graphene in Reference [22]. To model the incident ion and graphene interactions, the Ziegler-Biersack-Littmark (ZBL) potentials [23] is used, which contains a short-range repulsive force between all pairs of atoms to better describe the impact of atoms. The metal substrate is modeled by the embedded atom model (EAM) potential via LAMMPS. Meanwhile, the interaction between the graphene and the substrate is assumed to be van der Waals (vdW) type, and is modeled by the Lennard-Jones (LJ) potential V_ij_(r) = 4εij[(σ/r)^12^ − (σ/r)^6^], where i = C, j = Cu, and r is the interatomic distance. The values of parameters ε_ij_ and σ are taken from Ref. [24]. In the MD simulation, Berendsen temperature control technique is employed at the four borders of graphene and copper substrate in x- and y- directions. Additionally, the simulation system adopts the microcanonical ensemble (NVE, Eden Prairie, MN, USA). After the system is allowed to relax at room temperature of 300 K for 50 ps, the incident ion is introduced into the cell. Further, when the collisions are over, the temperature of system reduced to 300 K again. Crystallographic orientation of the substrate is (111). The time step after the introduction of irradiation is set in stages: From 0.01 fs to 1 fs. When the temperature of the system is lower than 500 K and the structure of the system no longer changes significantly, the simulation ends.

## 3. Results and Discussion

**Figure 1:**
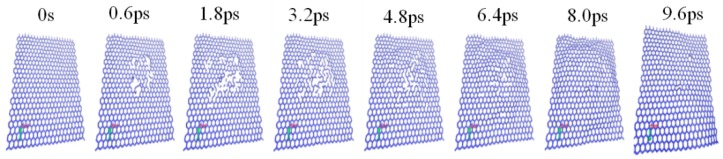
The temporal evolution of the irradiation-defects in suspended SLG system with irradiation energy of 500 eV.

Figure 1 illustrates the atomic configurations of suspended SLG system as the function of time after irradiation with 500 eV energy. It can be observed that the C–C covalent bonds are broken when the ion-collide with graphene, and the obvious amorphous region can be seen. However, the collided carbon atoms are still near the surface of materials. As the surface temperature of graphene gradually decreases, these carbon atoms reformed sp^2^ hybridization, and the previously formed defects disappear. Meanwhile, the surface of graphene tends to become flat. It is demonstrated that the main source of radiation-defects in graphene is knock-on atoms. Therefore, in this work, defect is defined as the carbon atom displaced more than 5 Å from its original lattice site.

**Figure 2:**
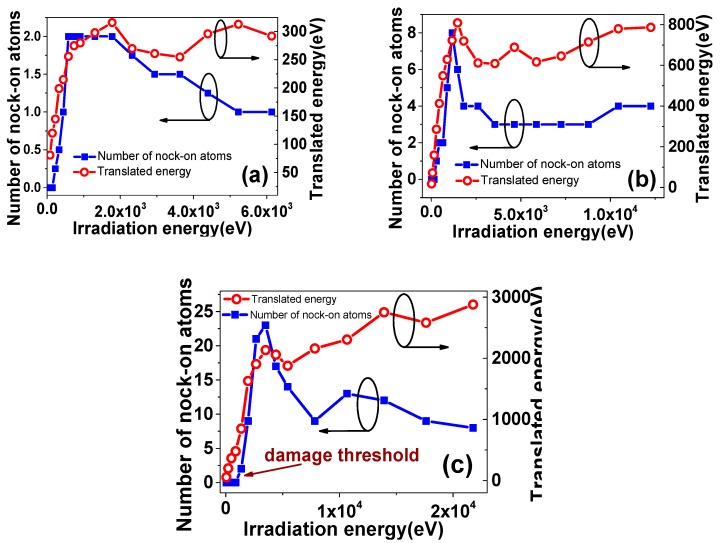
The number of knock-on atoms and translated energy as a function of irradiation energy: the maximum irradiation energy are: (**a**) 6.13 keV; (**b**) 12.26 keV; and (**c**) 21.77 keV.

When the target materials are bombarded, the incident ion beam releases energy to graphene by direct collisions with lattice carbon atoms and by interaction with electrons in the sheets. Figure 2 shows the number of knock-on atoms as a function of irradiation energy. As can be seen from Figure 2c, there existed damage threshold, below which no defect could be found in the graphene. In this work, the influence of different energy deposition model on the defect production in SLG is also checked. The results show that the damage threshold increases with the increase of the energy deposition, as shown in Figure 2. Meanwhile, it is noteworthy that, with the increase in irradiation energy, the number of knock-on atoms increases and starts to fall when the magnitude of irradiation energy is just 10^3^ eV. Additionally, the simulation results of suspended case are consistent with References [6,7].

**Figure 3:**
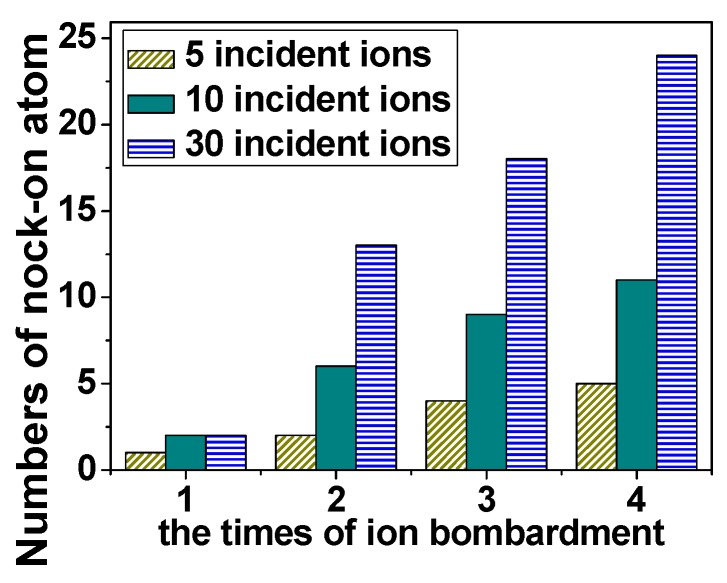
The number of knock-atoms as a function of times of ion bombardment. The numbers of incident ions are 5, 10, and 30, and the kinetic energy for each incident ion is 45 eV.

In order to further clarify how the SLG is damaged with increasing irradiation energy, the translated energy (transfer of kinetic energy from incident beams to carbon atoms) is analyzed, as also shown in Figure 2. At the beginning of irradiation, the growth behavior of the knock-on atoms has the similar tendency with translated energy. It can be further demonstrated that whether the carbon atoms are pulled out and what the number of knock-on atoms reaches are closely related to the kinetic energy, which the graphene actually obtains. Further, Figure 3 shows the number of knock-on atoms as a function of the incident dose (number of the total incident ions) varying from 5 to 30 after irradiation with one, two, three, and four-pulse. It can be clearly seen that the growing number of knock-on atoms with the increasing times of ion bombardment due to increases in the translated energy.

**Figure 4:**
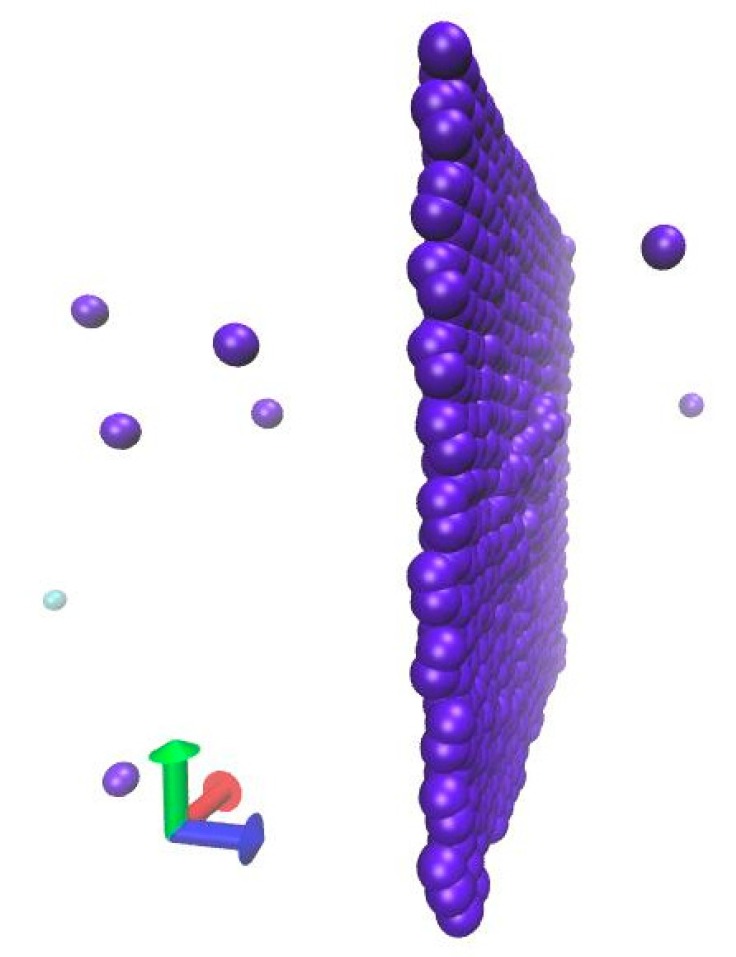
The collision between incident ions and graphene, when irradiation energy is 10 keV and the number of incident ions is 30.

When the ion energy further becomes larger, the number of knock-on atom falls, but in the meantime, the translated energy continues to grow, as shown in Figure 2. That is because the kinetic energy the graphene obtaining is not evenly distributed on the carbon atoms, which are located near the collision area. In detail, it is important to mention that, for this small system size, the incident ion can produce a bucking in the graphene layer. But when the ion energy is large enough, it is obvious that, as the incident ion flowed in one direction, a part of collided atoms move in the opposite direction, which can be seen in Figure 4. Additionally, those atoms will happen to cascade collision with the subsequent incident ion. That led to the uneven distribution of kinetic energy, which cause the number of atoms to become less coordinated with translated energy. Therefore, it can be deduced that the both actual transmission and distribution of kinetic energy play important roles in irradiation-defects forming process together.

**Figure 5:**
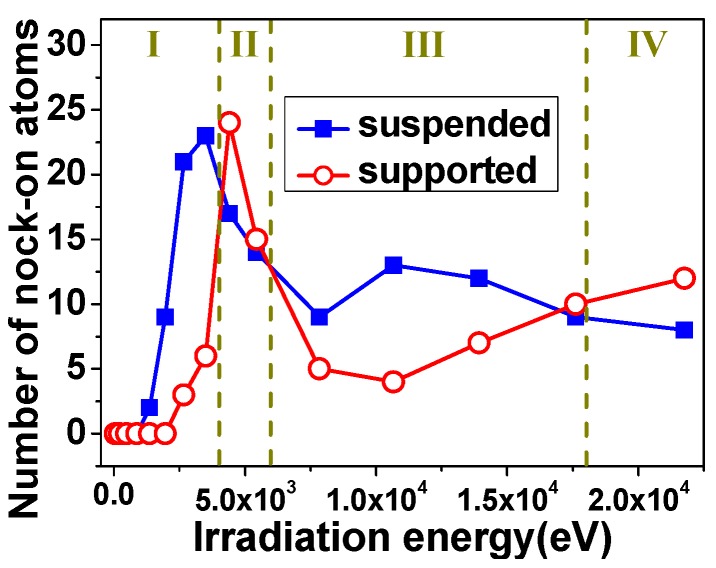
The number of knock-on atoms as a function of irradiation energy with 30 incident dose: Solid dots for suspended SLG and open dots for graphene grown on copper substrates.

**Figure 6:**
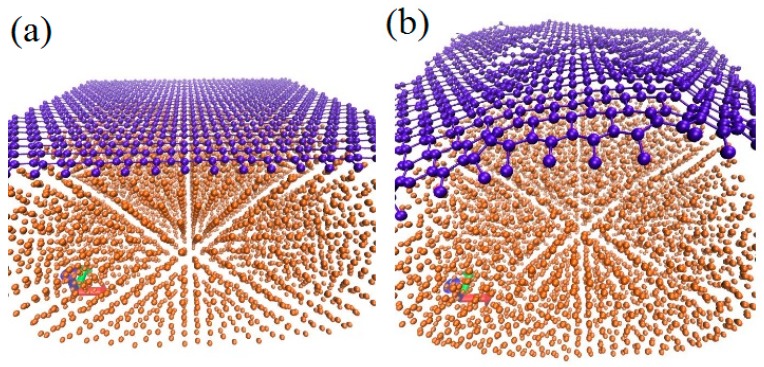
The atomic configurations of graphene-copper systems: (**a**) pre-irradiation and (**b**) after ion bombardment with 900 eV irradiation energy.

Figure 5 shows the comparison of defect numbers between suspended SLG and SLG grown on copper substrate with 30 incident dose. As can be seen, the minimum value needed to generate defects in supported graphene is higher than 2.67 keV, which is higher than the threshold value for track formation in copper. For a suspended graphene sheet, the value is determined to be 1.36 keV. This simulation results indicate that the present of copper substrate increases the damage threshold of graphene. These variation tendencies in numbers of knock-on atom for supported SLG may present four stages depending on the magnitude of incident energy. When the irradiation energy is less than 4 keV, there may exist irradiation damage. However, the carbon atoms are not pulled out far due to the influence of substrate, and collided with substrate to loss kinetic energy, which is still nearby the surface of graphene. Meanwhile, the high temperature produced by the collision between ion beam and graphene promote these free carton atoms sp^2^ hybridizing, thereby reducing damage production. Figure 6 shows uplift of the graphene surface in the opposite direction of incident ion after irradiation, which is different from the suspended graphene system, as shown in Figure 3. Thus, it is proved that the free carbon atoms can be rebounded by the substrate.

Due to the good mechanical property of graphene, more energy is required to break the C–C bond than to remove the Cu atom from substrate. When the irradiation energy further increases (i.e., 4–6 keV), the incident ion not only cause structural damage to graphene and penetrates the graphene to excite the Cu atom, but also move back and forth between these two layers to transfer almost all kinetic energy. Thus, more irradiation defects are produced in the supported SLG than that in the suspended SLG, as shown in Figure 5, and this damage is difficult to restore.

However, when the irradiation energy become much larger (i.e., 6–18 keV), the incident ion penetrates the graphene and moves into the substrate. That leads to the interaction between ion and graphene only once. Unlikely suspended graphene case, the knock-on atoms can lose a part of energy to substrate, and is weakened to escape from the graphene system. Hence, the irradiation damage of supported SLG is less than that of suspended SLG with the higher ion energy, as shown in Figure 5. It can be demonstrated that the presence of copper substrate would have a beneficial effect for the reconstruction of defective graphene. Nevertheless, when the irradiation energy is larger, more than 18keV, and the track formation in copper resulting in sputtered Cu atoms [7], which can collide with carbon atoms in graphene and finally lead to the defect formation in the graphene lattice. This is equivalent to the second irradiation on graphene layer. Additionally, the “rebound” phenomenon arises from the number of knock-on atoms in this larger deposition energy.

## 4. Conclusions

Classical molecular dynamics simulations have been carried out to study the defect forming process of SLG irradiated by ion beams. It is found that the actual transmission and distribution of kinetic energy from incident ion play important roles in defects forming the process together. On one hand, the more kinetic energy obtained, the more knock-on atoms formed, on the other hand, the uneven distribution of kinetic energy caused by the collision cascade constraints on the growth for the number of iradiation-defects. In addition, the influence of copper substrate on the irradiation effects of SLG are also analyzed. The simulation results indicate that the damage of supported graphene is due to the synergic effects from direct collision and indirect impact from the substrate. Thus, it is further suggested that the presence of the copper substrate increases the damage threshold of graphene.
